# A quantitative assessment of the evolution of cerebellar syndrome in children with phosphomannomutase-deficiency (PMM2-CDG)

**DOI:** 10.1186/s13023-017-0707-0

**Published:** 2017-09-15

**Authors:** Natalia Lourdes Serrano, Victor De Diego, Daniel Cuadras, Antonio F. Martinez Monseny, Ramón Velázquez-Fragua, Laura López, Ana Felipe, Luis G. Gutiérrez-Solana, Alfons Macaya, Belén Pérez-Dueñas, Mercedes Serrano, CDG Spanish-Consortium, Ma Concepción Miranda, Francisco Carratala, M. Pilar Póo, Bernabé Robles, María L. Couce, Marisa Girós, Laura Gort, Raquel Montero, Rafael Artuch, Celia Pérez-Cerdá, Jordi Muchart, Belén Pérez

**Affiliations:** 1 Neuropediatric, Radiology and Clinical Biochemistry Departments, Hospital Sant Joan de Déu, Barcelona, Spain; 2 U-703 Centre for Biomedical Research on Rare Diseases (CIBER-ER), Instituto de Salud Carlos III, Barcelona, Spain; 3 Pediatrics Department, Hospital Garrahan, Buenos Aires, Argentina; 4 Statistics Department, Fundació Sant Joan de Déu, Barcelona, Spain; 5 Pediatric Institute for Genetic Medicine and Rare Diseases, Hospital Sant Joan de Déu, Barcelona, Spain; 6 Pediatric Neurology Department, Hospital Universitario La Paz, Madrid, Spain; 7 Unit of Child Neurology, Department of Pediatrics, Hospital Infantil Universitario Niño Jesús de Madrid, Madrid, Spain; 8 Grup de Recerca en Neurologia Pediàtrica, Institut de Recerca Vall d’Hebron, Universitat Autònoma de Barcelona, Secció de Neurologia Pediàtrica, Hospital Universitari Vall d’Hebron, Barcelona, Spain; 9 Neurology Department, Hospital Sant Joan de Déu, Passeig Sant Joan de Déu, 2, 08950 Esplugues, Barcelona Spain

**Keywords:** Cerebellum, Congenital disorders of glycosylation, Developmental disorders, Gait disorders/ataxia, ICARS, MRI

## Abstract

**Background:**

We aim to delineate the progression of cerebellar syndrome in children with phosphomannomutase-deficiency (PMM2-CDG) using the International Cooperative Ataxia Rating Scale (ICARS). We sought correlation between cerebellar volumetry and clinical situation. We prospectively evaluated PMM2-CDG patients aged from 5 to 18 years through ICARS at two different time points set apart by at least 20 months. We reviewed available MRIs and performed volumetric analysis when it was possible.

**Results:**

From the eligible 24, four patients were excluded due to severe mental disability (*n* = 2) and supratentorial lesions (*n* = 2). Two different ICARS evaluations separated by more than 20 months were available for 14 patients showing an improvement in the cerebellar syndrome: ICARS1: 35.71 versus ICARS2: 30.07 (*p* < 0.001). When we considered time, we saw an improvement of 2.64 points in the ICARS per year with an SD of 1.97 points (*p* < 0.001). The ICARS subscales results improved with time, reaching statistical significance in “Posture and gait” (*p* < 0.001), “Kinetic functions” (*p* = 0.04) and “Speech abnormalities” (*p* = 0.045). We found a negative correlation between the ICARS results and total cerebellar volume (*r* = −0.9, *p* = 0.037) in a group of five patients with available volumetric study, meaning that the higher the ICARS score, the more severe was the cerebellar atrophy.

**Conclusions:**

Our study shows a stabilization or mild improvement in the cerebellar functions of paediatric PMM2-CDG patients despite cerebellar volume loss. ICARS is a valid scale to quantify the evolution of cerebellar syndrome in PMM2-CDG patients. The availability of ICARS and other reliable and sensitive follow-up tools may prove essential for the evaluation of potential therapies.

**Electronic supplementary material:**

The online version of this article (10.1186/s13023-017-0707-0) contains supplementary material, which is available to authorized users.

## Background

Phosphomannomutase deficiency (PMM2-CDG), is caused by mutations in *PMM2* (MIM *#601,785), and is the most frequent congenital disorder of N-linked glycosylation [1]. Patients with PMM2-CDG develop a cerebellar syndrome with axial and peripheral ataxia, abnormal eye movements, dysarthria, and cognitive deficits which cause long-term disability [2].

Brain MRIs and neuropathological studies show a selective involvement of the cerebellum. The increase in inter-folia spaces and the cortical brightness observed on MRIs are probably the consequence of the neuronal loss and secondary gliosis observed in pathological studies [3–5]. The atrophy is progressive and greater during the first decade of life and is present in the majority of patients, even in those clinically less affected [6, 7]. However, clinicians normally find stabilization or even an amelioration of cerebellar symptoms through the first and second decade of life (personal observation). Recently, the International Cooperative Ataxia Rating Scale (ICARS) has been validated for children suffering from PMM2-CDG [8]. However, no follow-up studies have been published quantifying the evolution of cerebellar syndrome using a clinical scale.

In our study, we aimed to quantify the evolution of cerebellar syndrome in PMM2-CDG patients across the paediatric age span using ICARS. We also analysed cerebellar volume on neuroimaging studies to find a relationship between the severity of the cerebellar syndrome and the cerebellar volumetric evaluation.

## Methods

Our study was designed as a prospective and observational study including Spanish patients that were diagnosed with PMM2-CDG and followed in tertiary care hospitals included in the Spanish CDG Network. Patients were recruited and followed from March 2014 to December 2016. The patient’s cerebellar syndrome was evaluated using ICARS at two different times of evolution with a minimum interval of 20 months in between evaluations.

The inclusion criteria were as follows: children and adolescents with genetically confirmed PMM2-CDG aged 5 to 18 years, at the time of assessment.

The exclusion criteria were any of the following: severe cognitive impairment or behavioural problems that prevented complete administration of the scale, the co-occurrence of supratentorial processes, the presence of a stroke-like episode during the study period or the intake of any non-validated therapy.

Early physiotherapy intervention programs were not an exclusion criterion. Medical problems which could have modified the results (such us an orthopaedic surgery), were registered.

The same child neurologist rated the PMM2-CDG patients with the ICARS in the different medical centres. The examinations were videotaped following a standardized protocol for educational and revision purposes. ICARS involves a 100-point rating scale with higher scores denoting more evident clinical abnormalities [9]. ICARS includes subscores for posture and gait (0–34), kinetic functions (0–52), speech abnormalities (0–8) and oculomotor function (0–6).

Biochemical and molecular studies were carried out in all the PMM2-CDG patients enrolled. Genetic analysis was performed at the Centre of Diagnosis of Molecular Diseases, Autonomous University of Madrid (CEDEM-UAM) in Spain.

The available MRIs were reviewed, but no new radiological explorations were requested. The brain MRI examinations included T1 and T2-weighted, diffusion-weighted and FLAIR sequences. For the volumetric analysis, SPGR 3D T1-axial images were transferred to Philips IntelliSpace Portal 4.0 software, [http://www.mea.philips.com/healthcare/product/HCNOCTN281/intellispace-portal-7] where a 3D brain image was automatically generated, as previously reported [6].

Ethical permission for the study was obtained from the Research & Ethics Committee of the HSJD. Parents gave their written informed consent and children/adolescents gave their assent. Samples were obtained in accordance with the Helsinki Declaration of 1964, as revised in October 2013 in Fortaleza, Brazil.

We performed our statistical analysis using SPSS 19.0 software (Armonk, NY: IBM Corp.) In order to compare the results of ICARS1 (first scale evaluation) to ICARS2 (second scale evaluation), and between the different subscales, we used the T Student Test. *P*-values of less than 0.05 were considered significant. To analyse the correlation between time, age and ICARS results, we used Pearson’s correlation. We studied the correlation between ICARS and the cerebellar volumes by calculating Spearman’s correlation (non-parametric). All the statistical tests were two-sided.

## Results

### ICARS assessment over time

Forty-three PMM2-CDG patients were registered in our data base. From those, nine were older than 18 years and seven were younger than 5 years, at the time of assessment, and were discarded. Three patients from the database died during the first 2 years of life.

From the eligible sample of 24, four (16.7%) were excluded due to severe intellectual disability (*n* = 2) and supratentorial lesions (one due to cranial haemorrhage and the other due to hydrocephaly and ischemic lesions secondary to cardiac arrest).

Of the 20 remaining patients, two different ICARS evaluations separated by more than 20 months were available for 14 patients.

Our final sample included eight girls and six boys, aged 5 years 6 months to 16 years at the time of the first ICARS evaluation (mean age: 9 years 4 months, standard deviation (SD): 3 years 9 months). The time period between ICARS1 and ICARS2 evaluations was 20 to 34 months (mean: 26.3 months, SD: 4.8 months). One patient underwent hip surgery 10 months before the second ICARS evaluation, but she was not excluded from the study.

**Fig. 1:**
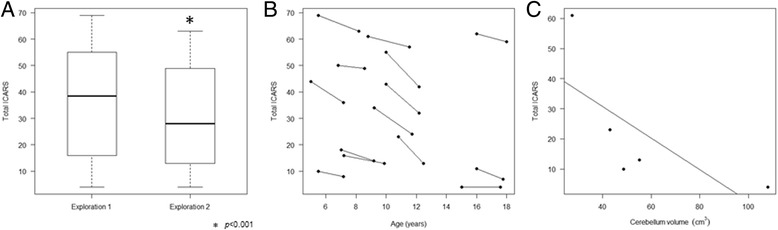
ICARS results in PMM2-CDG patients during the first and the second evaluation. **a** Boxplot representing ICARS1 (mean 35.71) compared to ICARS2 (mean 30.07) (*p* < 0.001) showing an improvement of ICARS results of 5.64 points in the study period. **b** This figure represents the decrease of ICARS over time for each patient. Points connected by lines show the ICARS measurements of the same patient. **c** Total ICARS is negatively correlated with cerebellar volumetry (*r* = 0.9, *p* = 0.037) for a group of five patients, in which volumetric study and ICARS were available between 5 and 8 years (68 to 105 months of age)

The ICARS1 values were higher than the ICARS2 (35.71 (SD 21.98) versus 30.07 (SD 21.06), (*p* < 0.001)) which shows an improvement of cerebellar symptoms of 5.64 points in the study period (SD: 4.10) (Fig. 1a and b). All the patients improved their functional cerebellar capabilities with the exception of one case with a very mild phenotype who retained an ICARS of 4/100 (range of improvement: 0 to 13 points). We show elements of the two ICARS evaluations of one patient in an additional movie file (see Additional file 1). The decrease of ICARS over time for each patient is represented in Fig. 1b. Table 1 shows molecular studies and ICARS results for every patient.

**Table 1:** Patients’ characteristics and ICARS evolution

ID	Gender/ Age ^a^	Molecular findings	Period of evaluation	ICARS1	ICARS2	Improvement	MRI
1	F/ 5 yr. 6 mo	c.368G > A/ c.722G > C	20 mo	10	8	2/100	CA progression
2	M/ 10 yr. 9 mo	c.484C > T/ c.523 + 3A > G	20 mo	23	13	10/100	CA progression
3	F/ 8 yr. 9 mo	c.338C > T/ c.353C > G + c.550C > A	33 mo	61	57	4/100	CA progression
4	F/ 9 yr. 2 mo	c.470 T > C/ c.484C > T	30 mo	34	24	10/100	Only one MRI available. CA.
5	M/ 15 yr	c. 278A > C/ c.422G > A	31 mo	4	4	0/100	CA progression
6	F/ 5 yr. 6 mo	c.338C > T/ c.338C > T	32 mo	69	63	6/100	CA progression
7	F/ 7 yr. 2 mo	c.470 T > C/ c.722G > C	32 mo	16	13	3/100	Only one MRI available. CA
8	F/ 16 yr	c.367C > T/ c.458 T > C	24 mo	62	59	3/100	Only one MRI available. CA.
9	M/ 5 yr. 1 mo	c.415G > A/ c.722G > C	26 mo	44	36	8/100	CA progression
10	M/ 7 yr	c.95 T > G/ c.422G > A	26 mo	18	14	4/100	Only one MRI available. CA.
11	F/ 10 yr	c.710C > T/ c.640-9 T > G	26 mo	55	42	13/100	Only one MRI available. CA.
12	F/ 10 yr. 3 mo	c.338C > T/ c.626A > G	26 mo	43	32	11/100	CA progression
13	M/ 16 yr. 2 mo	c.710 C > G/ c.710 C > G	21 mo	11	7	4/100	CA progression
14	M/ 6 yr. 8 mo	c.422G > A/ c.710C > T	21 mo	50	49	1/100	CA progression
Mean / SD / Range	9 yr. 4mo / 3 yr. 9 mo / 5 yr. 6 mo-16 yr. 2 mo		26.3 mo / 4.8 mo / 20–33 mo	35.71 / 21.98 / 4–69	30.07 / 21.06 / 4–63	5.64 / 4.10 / 0–13	

*M* male, *F* female, *yr.* years, *mo* months, *CA* cerebellar atrophy, *SD* standard deviation

^a^Age at ICARS1 evaluation


Additional file 1:This video includes elements of the ICARS assessment of Patient 4. The video has four sections including the four ICARS subscales: “Posture and Gait disturbance”, “Kinetic functions”, “Speech disorders” and “Oculomotor disorders”. For every manoeuvre the first and the second explorations are consecutive in the video. (M4V 277427 kb)


To express the differences in time between both ICARS results, we used “years” as our unit of measurement as the period of time between the two evaluations was not exactly the same in all patients. On average, there was an improvement of 2.64 points per year with a SD of 1.97 points (*p* < 0.001), with a range of improvement from 6.02 (in a girl with an initial ICARS of 55, followed 26 months) to no improvement at all.

No correlations were observed between the annual difference and the age of the patients at assessment. There was no statistically significant correlation between the annual difference and the magnitude of ICARS1 (*r* = 0.21, *p* = 0.48).

The ICARS subscores (posture and gait (P), kinetic functions (K), speech abnormalities (S) and oculomotor function (O)) were evaluated independently. All the subscales improved with time, and reached statistical significance in posture and gait (ICARS1-*P* = 13.36 vs ICARS2-*P* = 10.50; *p* < 0.001), kinetic functions (ICARS1-K = 17.36 vs ICARS2-K = 15.29; *p* = 0.04) and speech abnormalities (ICARS1-S = 3.36 vs ICARS2-S = 3.07; *p* = 0.045), but had a non-statistically significant trend in oculomotor function (ICARS1-O = 1.86 vs ICARS2-O = 1.36; *p* = 0.105).

### Neuroimaging assessment

Twenty-three MRI images were available from the 14 patients included our study. All the patients had undergone at least one MRI before the first ICARS evaluation, generally during the first months or years of life. Nine patients underwent a second MRI that showed cerebellar atrophy progression (Table 1). For six of them, the follow-up MRI was obtained between both ICARS evaluations. The time between the two MRI images was very heterogeneous; therefore no statistical analysis could be drawn comparing radiological and clinical evolution.

For a group of five patients (Patients 1, 2, 3, 5, and 7), conventional MRI and volumetric cerebellar studies were obtained during the period when ICARS were assessed. The correlation for this small group between the cerebellar volumetric analysis and clinical phenotypes is represented in Fig. 1c, and depicts a negative correlation between the ICARS score and total cerebellar volume (*r* = −0.9, *p* = 0.037). Additional file 2 shows detailed molecular, radiological and clinical characteristics of three of those patients.

## Discussion

PMM2-CDG is a rare multisystem disease with an extremely variable phenotype targeting almost every organ in the human body. In the long term, the neurological phenotype is the main cause of disability, cerebellar syndrome being the primary neurological finding [1, 2]. However the natural history of the neurological impairment, including a quantification of the cerebellar syndrome, is largely unknown.

Our study shows a mild global improvement in cerebellar functions at the paediatric age. The improvement is more evident in “posture and gait” and “kinetic functions”. This fact may be explained by two reasons: firstly, fine and gross motor skills change significantly during the neurodevelopment of all children, as evidenced by the greater improvement in these areas in normal developing children [10]; and secondly, the “posture and gait” and “kinetic functions” subscores have more weight on the global scale, with seven items being scored from one to four points or more, as compared to the “speech abnormalities” and “oculomotor disorder” subscores, which are only evaluated by three items scored from one to three points.

One of the potential limitations in our study is the capability of patients to follow ICARS instructions. As previously reported [8], most PMM2-CDG patients completed the ICARS test despite intellectual disability. Therefore, the use of ICARS in the follow-up of PMM2-CDG paediatric patients is suitable in most of the cases. From the eligible 24, only two were excluded due to severe mental disability. Moreover, facilitating comprehension of the different manoeuvres by training the patient immediately prior to evaluation may help the procedure in younger patients.

Another limitation of our study is the heterogeneity of the sample. Due to the rarity of PMM2-CDG and the sample size, the study group of patients was not stratified following age and clinical severity criteria for the statistical analysis. The stratification and subgroup analysis of larger samples will raise more specific conclusions in terms of ICARS evolution.

The non-cerebellar aspects of the neurological spectrum of PMM2-CDG patients can act as potential confounders to ICARS assessment (muscle weakness, peripheral neuropathy, extracerebellar muscle hypotonia, and cognitive impairment) [8], however, some of them alter the final result all over the time in subsequent evaluations of the same patient, controlling the bias as the error is present through the examinations. Although, some non-cerebellar aspects can be progressive (such as peripheral neuropathy), and may be considered as a limitation in sequential ICARS evaluations.

Despite the progression of the cerebellar atrophy described previously [6, 7], our clinical experience (personal observation) and this study demonstrate that there is no progression of cerebellar symptoms in children affected by PMM2-CDG. This may be explained partly by the fact that fine and gross motor skills improve during neurodevelopment, as has been reported in previous studies, evaluating healthy children [9, 10]. In our sample, not only the younger patients but also the older ones, improved clinically. Probably, developmental maturation of motor skills, impact of different rehabilitation strategies, alternative strategies learnt with experience to control ataxia, and brain plasticity events may all account for this unexpected discrepancy.

Lastly, and possibly due to the limited sample size, our study failed to demonstrate greater improvements dependent on the age or severity of the phenotype. An analysis using stratification among ages and clinical severity in a larger sample could investigate whether younger patients or those most severely affected may show greater improvements with time.

Regarding a correlation between neuroimaging and cerebellar phenotype, we were not able to evaluate any MRIs at the exact time of ICARS assessment. Nevertheless, the follow-up MRIs corresponding to nine patients included in the study showed a progression of cerebellar atrophy over time, as it has been previously described [6, 7]. Of note, five patients underwent MRI and ICARS evaluation at similar ages and showed a negative correlation between cerebellar volume and ICARS severity, although, this needs confirmation in a larger sample.

## Conclusion

Our study shows a stabilization or mild improvement in cerebellar functions in paediatric PMM2-CDG patients above 5 years of age measured through ICARS, in contrast with cerebellar volume evolution [6, 7]. Our findings reinforce the importance of being cautious with the apparently degenerative processes of central nervous system during childhood. A better knowledge of the course of cerebellar syndrome in paediatric patients suffering from PMM2-CDG, and the availability of sensitive and reliable follow-up tool may prove essential for the evaluation of potential therapies in the future, particularly in younger children where there is no validated scale for cerebellar syndrome.

## Additional files

Additional file 2: Cerebellar volumetric study, molecular and clinical characteristics of Patients 3, 5 and 7. (TIFF 3555 kb)
