# Supplementary figures and images for: A quantitative assessment of the evolution of cerebellar syndrome in children with phosphomannomutase-deficiency (PMM2-CDG)
Source: Orphanet J Rare Dis. 2017 Sep 15;12:155. doi: 10.1186/s13023-017-0707-0 (PMC5602850; doi:10.1186/s13023-017-0707-0)

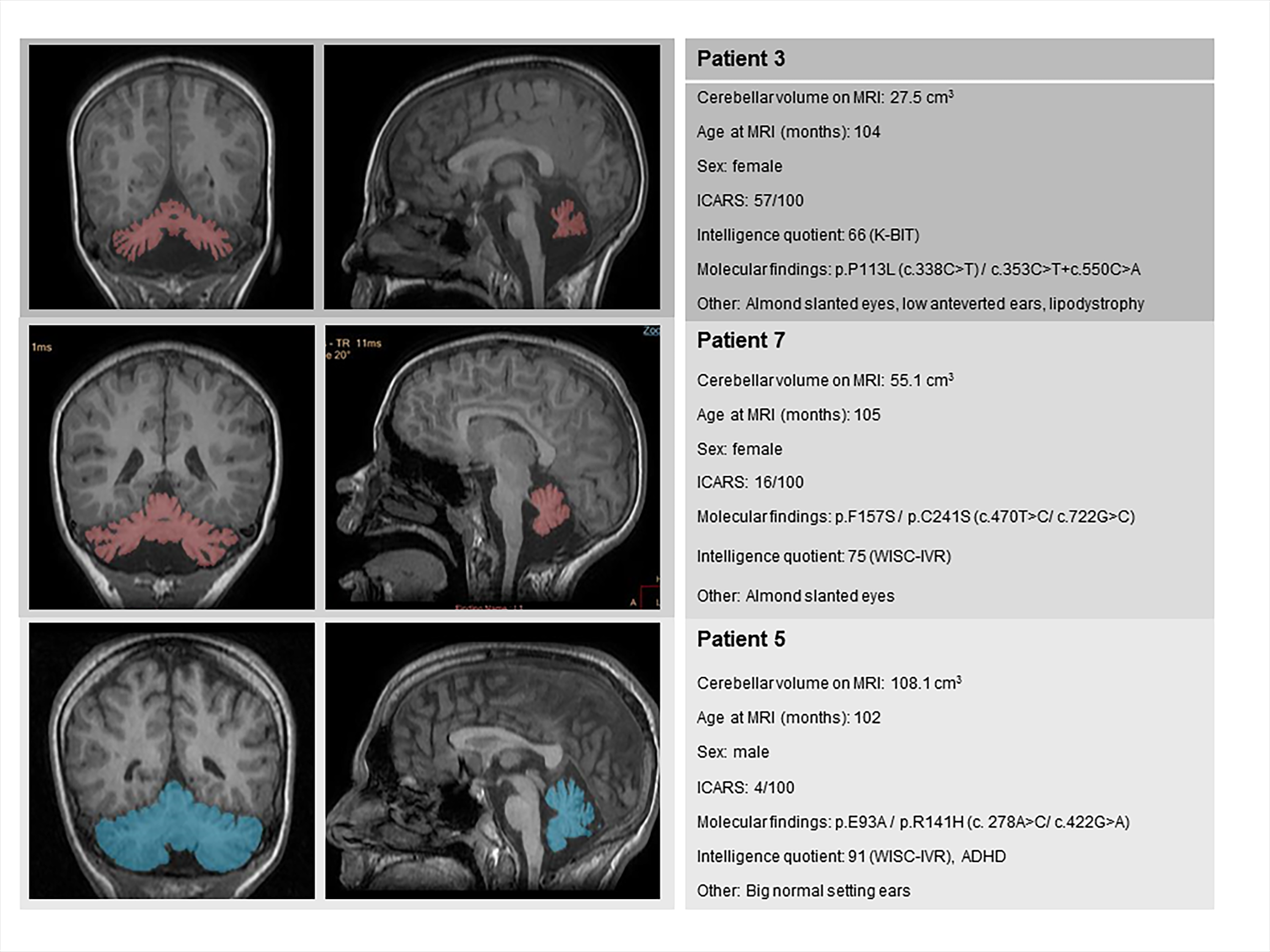

Supplement: Supplementary file 2 — Cerebellar volumetric study, molecular and clinical characteristics of Patients 3, 5 and 7. (TIFF 3555 kb) [file 13023_2017_707_MOESM2_ESM.tif]
